# Brief Post-Surgical Stress Management Reduces Pro-Inflammatory Cytokines in Overweight and Obese Breast Cancer Patients Undergoing Primary Treatment

**DOI:** 10.31083/j.fbl2705148

**Published:** 2022-05-07

**Authors:** Molly Ream, Estefany Saez-Clarke, Chloe Taub, Alain Diaz, Daniela Frasca, Bonnie B. Blomberg, Michael H. Antoni

**Affiliations:** 1 Department of Psychology, University of Miami, Coral Gables, FL 33146, USA; 2 Department of Medical Social Sciences, Northwestern University Feinberg School of Medicine, Chicago, IL 60611, USA; 3 Department of Microbiology and Immunology, University of Miami School of Medicine, Miami, FL 33124, USA; 4 Tumor Biology Program, Sylvester Comprehensive Cancer Center, Miami, FL 33136, USA; 5 Cancer Control Program, Sylvester Comprehensive Cancer Center, Miami, FL 33136, USA

**Keywords:** breast cancer, overweight, obese, inflammation, stress management, relaxation, cognitive behavior therapy

## Abstract

**Background:**

Overweight and obese (OW/OB) body mass index (BMI) is associated with greater inflammation and poorer outcomes in breast cancer (BC). Stress management interventions using cognitive behavioral therapy (CBT) and relaxation training (RT) have reduced inflammation in BC patients but have not been tested specifically in OW/OB patients undergoing primary treatment. We developed brief CBT and RT-based group interventions and tested their effects (vs time-matched Health Education [HE] control) on serum inflammatory cytokines (IL-6, IL-1*β* and TNF-*α*) in OW/OB vs normal weight (NW) BC patients during primary treatment. We hypothesized OW/OB women would show higher levels of inflammatory cytokines, and that stress management would decrease these cytokines more in OW/OB women than in NW women.

**Methods:**

Stage 0 – III BC patients were enrolled post-surgery and before initiating adjuvant therapy, were randomized to either 5 weeks of CBT, RT, or HE, and provided questionnaires and blood samples at baseline and 6-months. Serum cytokine levels were measured by ELISA. Repeated measures analysis of variance tested the interaction of condition by BMI by time in predicting cytokine levels over 6 months, controlling for age, stage, ethnicity, and income.

**Results:**

The sample (N = 153) majority was OW/OB (55.6%). We found differences in baseline IL-6 and IL-1*β* across BMI categories, with greater IL-6 (*p* < 0.005) and IL-1*β* (*p* < 0.04) in OW and OB vs NW women, but no difference between OW and OB women. There were no differences in baseline TNF-*α* among BMI groups. BMI category moderated the effect of brief stress management interventions on IL-6 changes over 6-months (*p* = 0.028): CBT/RT vs HE decreased IL-6 in OW/OB (*p* = 0.045) but not in NW patients (*p* = 0.664). There were no effects on IL-1*β* or TNF-*α*. Results could not be explained by differences in receipt of adjuvant therapy, prescription medications, or changes in physical activity.

**Conclusions:**

OW/OB women with newly diagnosed BC had significantly greater serum IL-6 and IL-1*β* than NW women post-surgery. Brief stress management delivered with primary treatment among OW/OB patients may reduce the increases in inflammatory markers known to accompany adjuvant treatments and could thus promote better outcomes.

**Clinical Trial Registration:**

NCT02103387.

## 1. Introduction

Breast cancer (BC) is the most common cancer for women worldwide [1]. Notably, obesity has been identified as a risk factor for BC and has been associated with higher rates of obtaining a BC diagnosis and worse prognosis [2,3]. The World Health Organization (WHO) and the National Institute of Health (NIH) utilize body mass index (BMI [kg/m^2^]) to define the categories of overweight (OW; BMI = 25 to 29.9 kg/m^2^) and obese (OB; BMI ≥30 kg/m^2^). Worldwide, more than 600 million adults were classified as obese in 2015 [4]. Importantly, OW/OB individuals are at higher risk of negative health outcomes, including being at higher risk of developing cancer [5,6], including breast cancer [7–11]. Excessive weight has been associated with worse prognosis and worse mortality rates for breast cancer [3]. Additionally, both pre- and post-menopausal OW/OB women have a greater likelihood of BC recurrence and mortality [12,13]. Being overweight or obese has also been linked with an elevated risk of obtaining an additional cancer diagnosis (e.g., in the previously unaffected breast or in a separate primary site) [14,15]. Finally, chemotherapy and endocrine treatments have been reported to be less likely to be effective in women with obesity [16–20].

The relationship between obesity and BC has been attributed to chronic adipose tissue inflammation which leads to a microenvironment favorable to cancer growth [21,22]. Accordingly, obesity has been consistently linked to greater inflammation [23–28]. High adiposity in OW/OB individuals is associated with dysregulated metabolic pathways [23], including increased secretion of pro-inflammatory cytokines [24–29] and adipokines [27,29]. This increased inflammation related to adipose tissue dysfunction has been linked to increased BC risk in OW/OB women [30]. Moreover, chronic inflammation and increases in pro-inflammatory cytokines tumor necrosis factor alpha (TNF-*α*) and interleukin-6 (IL-6), which both contribute to cell proliferation in breast adipose stromal cells [23], may be a possible mechanism through which obesity contributes to worse health outcomes in diagnosed BC patients [30–32].

Despite adiposity being a theoretically modifiable factor related to inflammation, a recent meta-analysis suggests that interventions targeting adiposity (i.e., physical activity, caloric restriction, weight management) do not significantly decrease inflammatory cytokines, including IL-6 and TNF-*α*, in BC survivors [33]. Another modifiable factor related to inflammation is stress [34]. The stress response involves release of stress hormones including norepinephrine, epinephrine, and cortisol, as well as increased secretion of proinflammatory cytokines [34–37]. Women newly diagnosed with BC have elevated levels of depression and anxiety [38], and thus are particularly susceptible to experiencing the downstream effects of the stress response. Notably, OW/OB women with BC are more likely to have a history of chronic stress [39]. Therefore, OW/OB women with newly diagnosed BC are highly susceptible to experience increased inflammation due to chronic and acute stress, as well as increased adiposity.

Stress management interventions (e.g., Cognitive-Behavioral Stress Management [CBSM]) have reduced distress and inflammatory markers in BC patients undergoing primary treatment [40–42]. CBSM is a 10-week group-based intervention that combines relaxation training (RT) and cognitive behavioral therapy (CBT) [43]. Among women recruited in the weeks after surgery for non-metastatic BC, CBSM has been shown to reduce depressive symptoms [42], anxiety and negative affect [41] and inflammatory markers including leukocyte gene expression for IL-1*β*, IL-6, and TNF-*α* [40]. CBSM has also been associated with improved overall survival and longer disease-free survival (DFS) over an 11-year median follow-up [44]. Here greater reductions in leukocyte inflammatory gene expression with CBSM during primary treatment predicted a longer 11-year median DFS [45].

In the interest of comparing briefer forms of stress management intervention in this population we created 5-week versions of group-based CBT and RT and compared them with an attention-matched Health Education (HE) condition in women undergoing primary treatment for BC. We found both CBT and RT conditions were associated with decreases in psychological distress [46] and inflammatory markers when compared to the HE group [47,48]. However, these interventions have not been tested specifically in OW/OB women with BC. Due to the potentially interacting factors of stress and adiposity leading to increased inflammation in OW/OB women [23–28], these stress management interventions may be particularly beneficial in this vulnerable population and may lead to improved health outcomes via a reduction in inflammatory markers. Therefore, this study examined whether OW/OB women initiating treatment for BC showed higher levels of pro-inflammatory cytokines (IL-6, IL-1*β* and TNF-*α*) than normal weight (NW) women in the weeks after surgery; and whether these brief interventions reduced levels of these cytokines over a 6-month period of primary treatment to a higher degree in OW/OB women vs NW women. We hypothesized that OW/OB women would reveal greater levels of inflammatory cytokines than NW women at study entry (2–10 weeks after surgery). We also hypothesized that OW/OB women would show greater decreases (or less increases) in cytokines over the 6-month period after CBT or RT as compared to HE.

## 2. Materials and Methods

### 2.1 Participants

Participants for the current analysis were women who enrolled in a prior randomized controlled trial testing two different 5-week group-based stress management interventions, CBT and RT, vs an attention-matched HE control, which took place from 2006–2014 (National Institutes of Health Clinical Trial NCT02103387). This study was approved by the Institutional Review Board at the University of Miami. Participants completed informed consent prior to completing study procedures.

Women aged 21–75 with non-metastatic BC who were between 2- and 10-weeks post-surgery were recruited from various cancer clinics in South Florida for this trial. Exclusion criteria were clinical interview-determined severe mental illness (i.e., untreated mania or psychosis) that would interfere with ability to participate in a group intervention format, initiation of adjuvant chemotherapy or radiation treatment prior to enrollment, a previous diagnosis of cancer, and lack of English fluency.

### 2.2 Procedures

Enrolled participants completed a battery of psychosocial surveys and provided a blood sample at baseline (T1), prior to randomization to study condition. Women then participated in one of the three assigned group interventions (see 2.3). Immediately after the final group session, participants were given a follow-up questionnaire (T2; approximately 6 weeks post-T1) to test their perceived stress management skills. Six months post-randomization (T3), participants completed a third questionnaire to assess health behaviors and provided a second blood sample.

### 2.3 Intervention Conditions

All three study conditions involved 5 consecutive, weekly 1.5 hours intervention sessions in groups of 3–7 participants. Group facilitators (total = 7) were doctoral students in clinical psychology who received training to conduct one or more of the three group interventions, and received weekly face-to-face supervision by one of the study investigators (MHA) in order to maintain the fidelity to each intervention protocol, minimize drift and prevent contamination across study conditions. In addition to weekly sessions, all participants were also given a workbook that included the information reviewed in each session. See Supplemental Material for further information regarding intervention content.

#### 2.3.1 Cognitive Behavioral Therapy

The CBT group condition was based on the cognitive-behavioral components of CBSM [43], which aims to teach adaptive coping within a cognitive-behavioral framework with emphasis on cancer-related stressors. Women in this condition were taught stress awareness, appraisal processes, cognitive restructuring, coping effectiveness, and interpersonal skills (i.e., assertiveness, anger management). Homework assignments were given for additional practice outside of group.

#### 2.3.2 Relaxation Training

The RT group condition was based on the relaxation components of CBSM [43], which aimed to reduce stress and anxiety by teaching relaxation and mindfulness techniques including diaphragmatic breathing, progressive muscle relaxation, imagery, and mindful meditation. The rationale of these practices for stress management was also provided in the group format via psychoeducation. Participants were provided with audio recordings of each technique to practice at home.

#### 2.3.3 Health Education

HE, the attention-matched control, covered educational information relevant to BC patients including symptom management, treatments and resources available, and tips for living a healthy life post-cancer diagnosis. The content of health education material was drawn from publically available sites sponsored by the American Cancer Society (ACS) and the National Cancer Institute (NCI) [49,50] and presented in PowerPoint slides in the weekly sessions. No information on stress management was provided in this control condition. This condition was included to control for facilitator attention and the presence of a supportive group similar to what is available through supportive care services at many cancer centers [51].

### 2.4 Measures

#### 2.4.1 Body Mass Index

Participants self-reported height in inches and weight in pounds at T1. Given the post-hoc nature of the current analysis, the fact that the study took place in community practices, and the time elapsed since the conduct of the trial, BMI data at follow-up was not obtainable. Baseline BMI was calculated by dividing weight by height squared and multiplying by the metric conversion factor of 703 [52]. Participants were then categorized per the Center for Disease Control and Prevention as normal weight (18.5–24.9 m/kg^2^), overweight (25.0–29.9 m/kg^2^), and obese (≥30 m/kg^2^) [52]. No participants in our sample met criteria for the underweight category (<18.5 m/kg^2^) [52]. For study analyses, we combined overweight (OW) and obese (OB) individuals to maximize power, such that the two categories for BMI were normal weight (NW; 18.5–24.9 m/kg^2^) and overweight/obese (OW/OB; ≥25 m/kg^2^). However, prior to combining the OW and OB categories, we conducted descriptive analyses and three-group analysis of variance (ANOVA) of cytokine levels across all three BMI categories at T1 and T3.

#### 2.4.2 Inflammatory Cytokines

At T1 and T3, non-fasted blood samples (35 mL) were obtained between 4:00–6:30 PM. by a licensed phlebotomist and serum was subsequently separated by centrifugation. Concentration of three pro-inflammatory cytokines, IL-1*β*, TNF-*α*, and IL-6, in serum were measured using enzyme-linked immunosorbent assay (ELISA) kits from Life Technologies (Camarillo, CA, USA). All assays were conducted by trained laboratory personnel. Blood draws were conducted at baseline (T1) and 6-month follow-up (T3) only, based on prior literature demonstrating that a longer stress management intervention yielded decreased inflammatory markers at 6-months [45].

#### 2.4.3 Perceived Stress Management Skills

The Measure of Current Status [53] was collected at T1 (baseline) and T2 (post-intervention) to measure perceived stress management skills (PSMS). We calculated a composite score of items on this measure capturing confidence in two CBT and two RT skills. This composite (*α* = 0.720) has been used in previous research assessing the relationship between stress management and inflammatory processes in BC patients [47]. See Supplemental Material for the questionnaire.

#### 2.4.4 Physical Activity

Physical activity was measured with a brief version of the 7-Day Physical Activity Recall Questionnaire [54], in which patients recalled the total number of minutes of moderate and vigorous physical activity (MVPA) completed over the past 7 days. This measure has been used to capture MVPA among cancer patients [55,56]. We measured MVPA at T1 and T3. See Supplemental Material for the questionnaire.

#### 2.4.5 Covariates

Covariates for study outcomes included age, stage of disease (0-III), household income, and ethnicity (Hispanic, non-Hispanic White, other). These variables were collected at baseline through self-report, with stage and age being verified with subsequent medical record review. These covariates were based on previous literature suggesting that age and disease stage are related to inflammatory markers [57], and because they have been controlled for in our past studies relating inflammation to stress processes in breast cancer patients undergoing primary treatment [47,58,59]. We also collected self-report data at T1 and T3 on prescription medications [antidepressants, anxiolytics (drugs relieving anxiety), pain medications, sleep medications], and adjuvant treatments received (chemotherapy/radiation) in the period leading up to T3. We also collected self-reported data at T1 on presence of medical comorbidities, including diabetes, myocardial infarction, peripheral vascular disease, and connective tissue disease.

### 2.5 Analytic Plan

All analyses were conducted using the Statistical Package for the Social Sciences (SPSS) Version 27 (IBM Corp. Armonk, NY, USA) [60]. Data were initially screened for skewness and kurtosis. Values of IL-6, IL-1*β*, and TNF-*α* were skewed at T1 and T3. As such, all cytokine values were log-transformed, achieving normality. Raw cytokine values (pg/mL) are reported in tables for interpretability.

First, to justify collapsing overweight and obese BMI groupings to the OW/OB category and collapsing the two active stress management conditions (CBT/RT), we conducted two three-group analysis of variance (ANOVA) tests: (1) between CBT, RT, and HE, and (2) between NW, OW, and OB weight groupings, each predicting both T1 and T3 cytokine levels.

After we justified collapsing these categories, preliminary analyses were conducted to determine whether intervention condition or BMI predicted inflammation or variables potentially related to inflammation at T1 and T3. We compared active (CBT/RT) vs control (HE) groups separately by BMI category for baseline cytokine levels, self-reported physical activity (MVPA) levels, income, age, days from surgery to baseline assessment, and group attendance using *t*-tests. Chi-square tests were also used to examine differences between study conditions by BMI category on categorical variables at T1 and T3 including ethnicity (T1 only), prescription medications (antidepressants, anxiolytics, pain medications, sleep medications), chemotherapy/radiation receipt, stage of disease, ER/PR status, type of surgery (mastectomy vs lumpectomy), and receipt of reconstructive surgery.

Third, we conducted a manipulation check to determine whether women receiving CBT/RT had improved stress management skills over the course of the intervention as compared to the HE control. To test whether intervention condition affected perceived stress management skills pre- to post-intervention, and whether this differed by BMI, we tested a two condition (CBT/RT vs HE) by two group (OW/OB vs NW) by two timepoint (T1, T2) repeated measures ANOVA (RMANOVA) on the PSMS composite score, controlling for previously stated covariates. RMANOVA uses listwise deletion for missing data.

To test our primary hypothesis that baseline BMI moderated the effect of intervention condition (CBT/RT vs HE) on inflammatory cytokines pre- to 6-months post-randomization, we tested a two condition (CBT/RT vs HE) by two group (OW/OB vs NW) by two timepoint (T1, T3) RMANOVA on each inflammatory cytokine (IL-6, IL-1*β*, and TNF-*α*). In the case of a significant three-way interaction, we investigated the simple two-way interactions of condition by time at the different levels of BMI.

Finally, to investigate one potential mechanism by which the interventions may decrease inflammation, we assessed whether the relationship between condition (CBT/RT vs HE) and the change in self-reported weekly hours of MVPA from T1 to T3 was moderated by BMI. To assess this, we tested a two condition (CBT/RT vs HE) by two group (OW/OB vs NW) by two timepoint (T1, T3) RMANOVA on MVPA weekly hours. We then tested whether the moderating effect of BMI on intervention effects on cytokine changes held after controlling for contemporaneous changes in MVPA. The two-tailed alpha level for all analyses was set at 0.05.

## 3. Results

**Fig. 1.**
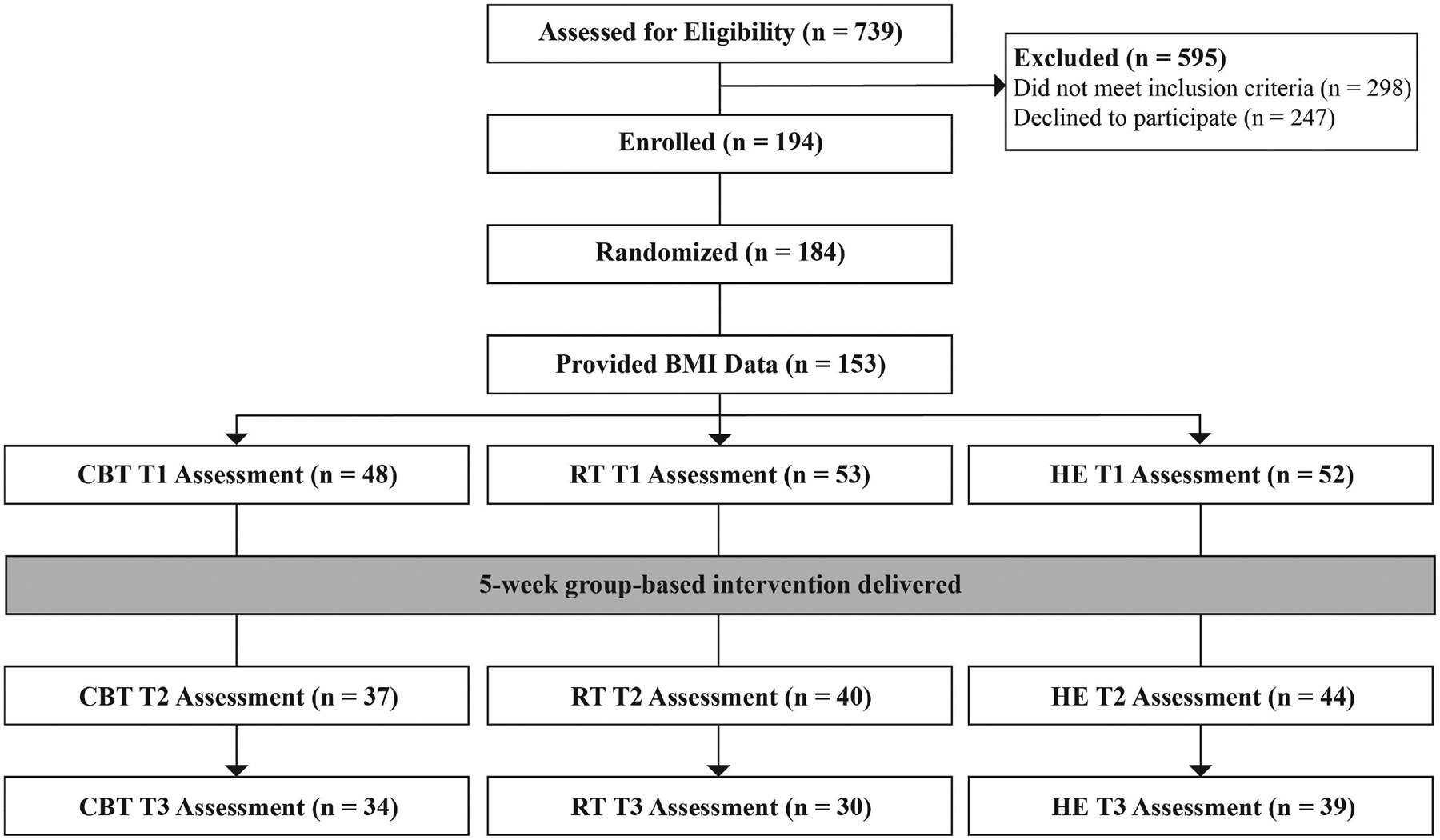
CONSORT flow diagram. CBT, Cognitive Behavioral Therapy; RT, Relaxation Training; HE, Health Education. Of the 153 women who provided BMI data, baseline cytokine data were available for 149 women.

In total, 739 women were assessed for eligibility, of which 194 met eligibility criteria and signed consent. Of these, 184 completed baseline procedures and were randomized. For the present analysis, we looked at the subsample of participants who self-reported weight and height data at baseline (N = 153; 83.2%). Women who declined to provide height and weight data did not differ from those who did on baseline levels of IL-6 (*t*(175) = −0.68, *p* = 0.498), IL-1*β* (*t*(176) = −0.65, *p* = 0.515), or TNF-*α* (*t*(176) = −0.27, *p* = 0.789), but were on average younger (*t*(180) = −2.31, *p* = 0.022) and had a lower income (*t*(180) = −2.45, *p* = 0.015). All 153 women completed baseline psychosocial questionnaires and 149 women provided blood samples at baseline. Post-intervention, 121 women completed the T2 perceived stress management skill measure (PSMS), and 103 gave a second blood sample at T3. See Fig. 1 for the CONSORT diagram.

### 3.1 Participant Characteristics

The sample (N = 153) was predominately middle-aged (*M* = 55.07, *SD* = 10.02), White (43.3%) and Hispanic (42.0%), with stage 1 (55.6%) disease. The majority (55.6%) fell within the OW/OB BMI (OW = 27.5%, OB = 28.1%), and 44.4% fell within the NW range. See Table 1 for full demographics and study variables by BMI classification and Table 2 for demographics and study variables by intervention condition.

### 3.2 Preliminary Analyses

We first compared cytokine values at baseline between intervention conditions (CBT, RT, HE) and between BMI groups (NW, OW, OB) using ANOVA. At baseline, there was a significant difference between intervention conditions in IL-1*β* (F(2,146) = 3.21, *p* = 0.043); however, post-hoc Fisher Least Squared Differences (LSD) tests showed only a marginally significant difference between RT and HE at baseline (*p* = 0.074) and no condition differences in baseline IL-6 (F(2,145) = 0.73, *p* = 0.484) or TNF-*α* (F(2,146) = 0.39, *p* = 0.681).

**Fig. 2.**
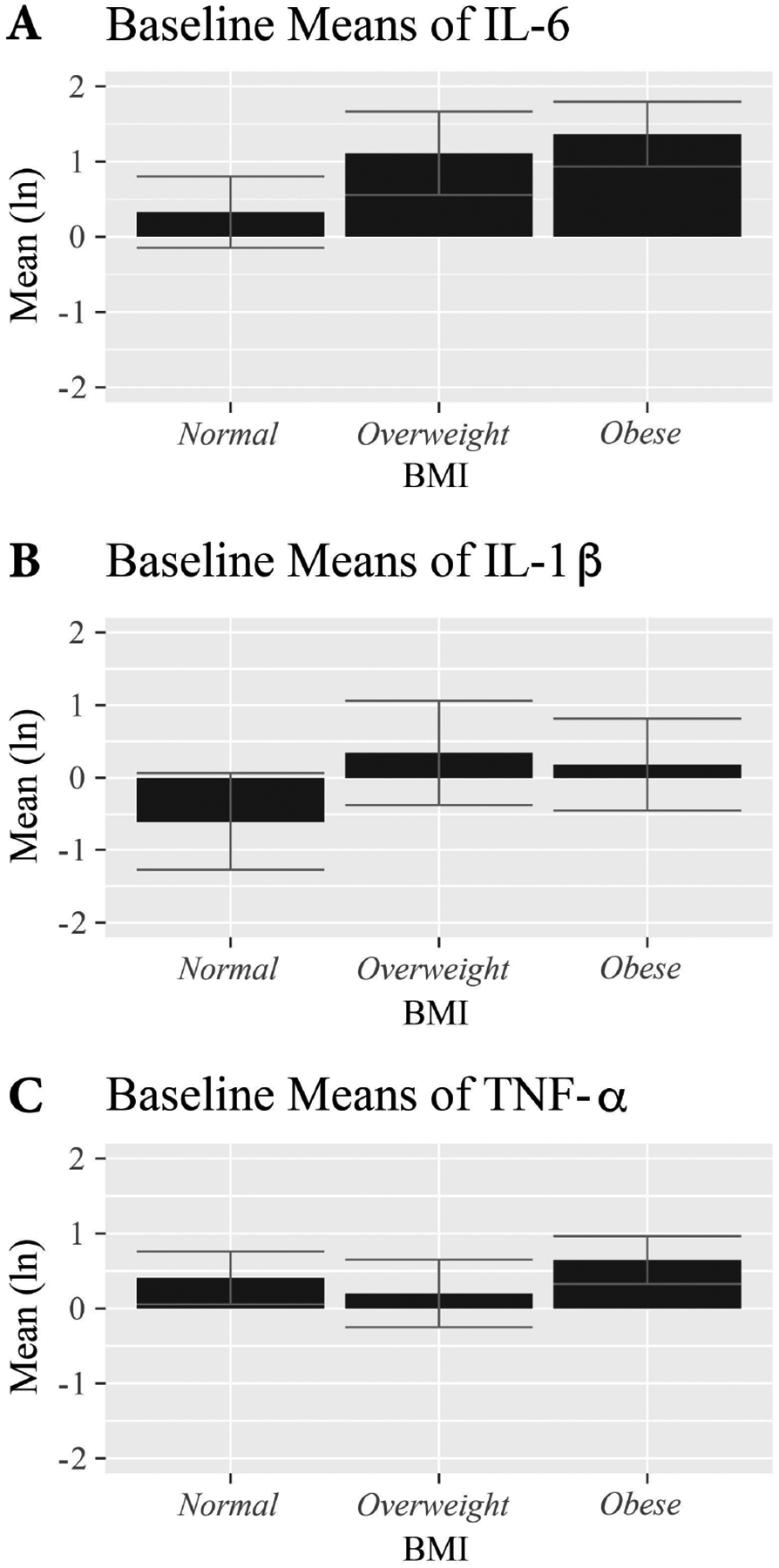
Mean baseline cytokine values by BMI category in breast cancer patients 2–10 weeks post-surgery and prior to initiating adjuvant chemotherapy or radiation. Cytokine levels are log-transformed to achieve normality. Error bars represent 95% confidence intervals. There was a significant overall difference in baseline IL-6 (F(2,145) = 8.45, *p* < 0.001), shown in Fig. 2A and IL-1*β* (F(2,146) = 3.89, *p* = 0.023), shown in Fig. 2B across BMI categories. There was significantly greater IL-6 in overweight (*p* = 0.005) and obese (*p* < 0.001) women vs normal weight women, but no statistical difference between overweight and obese women (*p* = 0.400). Similar results were observed for baseline IL-1*β* (normal weight vs overweight, *p* = 0.014; normal weight vs obese, *p* = 0.037; obese vs overweight, *p* = 0.704). There were no baseline differences in TNF-*α* values among BMI groups, shown in Fig. 2C.

As hypothesized there was a significant overall difference in baseline IL-6 (F(2,145) = 8.45, *p* < 0.001) and IL-1*β* (F(2,146) = 3.89, *p* = 0.023) across BMI categories. Post-hoc LSD pairwise tests showed significantly greater IL-6 in OW (*p* = 0.005) and OB (*p* < 0.001) women vs NW women, but no statistical difference between OW and OB women (*p* = 0.400). Similar results were observed for baseline IL-1*β* (NW vs OW, *p* = 0.014; NW vs OB, *p* = 0.037; OB vs OW, *p* = 0.704). There were no baseline differences in TNF-*α* values among BMI groups. See Fig. 2 for baseline means of each cytokine level across the three BMI categories. Given these results and to optimize power, BMI categories for overweight and obese (OW/OB) were combined as were the two active stress management intervention conditions (CBT/RT) for all subsequent analyses.

Only one participant (CBT/RT; OW/OB) had received radiation within the 3 weeks prior to T3, and only one participant (HE; OW/OB) received chemotherapy within the 3 weeks prior to T3. Neither receipt of chemotherapy (F(1,105) = 0.028, *p* = 0.868) nor radiation (F(1,105) = 0.754, *p* = 0.387) significantly predicted the change in IL-6. Similar results were observed for predicting change in IL-1*β* (chemotherapy receipt: (F(1,109) = 0.003, *p* = 0.959); radiation receipt: F(1,109) = 0.448, *p* = 0.505). Radiation receipt did significantly predict the change in TNF-*α* over time (F(1,109) = 8.82, *p* = 0.004), but chemotherapy receipt did not (F(1,109) = 1.02, *p* = 0.315). There were no differences in prescription medications (anxiolytics, antidepressants, sleep medications, or pain medications), ER/PR status, receipt of reconstructive surgery before T3, or surgery type (mastectomy vs lumpectomy) by BMI (Table 1) or intervention arm (Table 2). Self-reported comorbidities showed no differences between OB/OW vs NW women (*p* > 0.05) with the exception of diabetes, which showed greater prevalence in women classified as OW/OB (n = 11, 13.4%) compared to NW (n = 2, 2.9%) *χ*^2^(1) = 5.15, *p* = 0.023).

Most participants (61.4%) completed at least 4 out of 5 intervention sessions. There was no significant difference in session attendance between BMI categories (*p* = 0.085), with NW women attending an average of 4.29 out of 5 sessions as compared to OW/OB women attending an average 3.86 out of 5 sessions. A two condition (CBT/RT vs HE) by two group (OW/OB vs NW) by two timepoint (T1, T2) RMANOVA predicting the PSMS composite score was run as a manipulation check. There was a significant time by condition interaction (F(1,102) = 9.52, *p* = 0.003), such that women in the active CBT/RT conditions had greater increases in perceived stress management skills over time compared to women in the HE control condition. The three-way interaction was not significant (F(1,102) = 3.16, *p* = 0.078). Thus, assignment to either of the stress management interventions produced greater improvements in perceived stress management skills as compared to the HE condition irrespective of BMI group.

### 3.3 Main Analyses of Intervention Effects

**Fig. 3.**
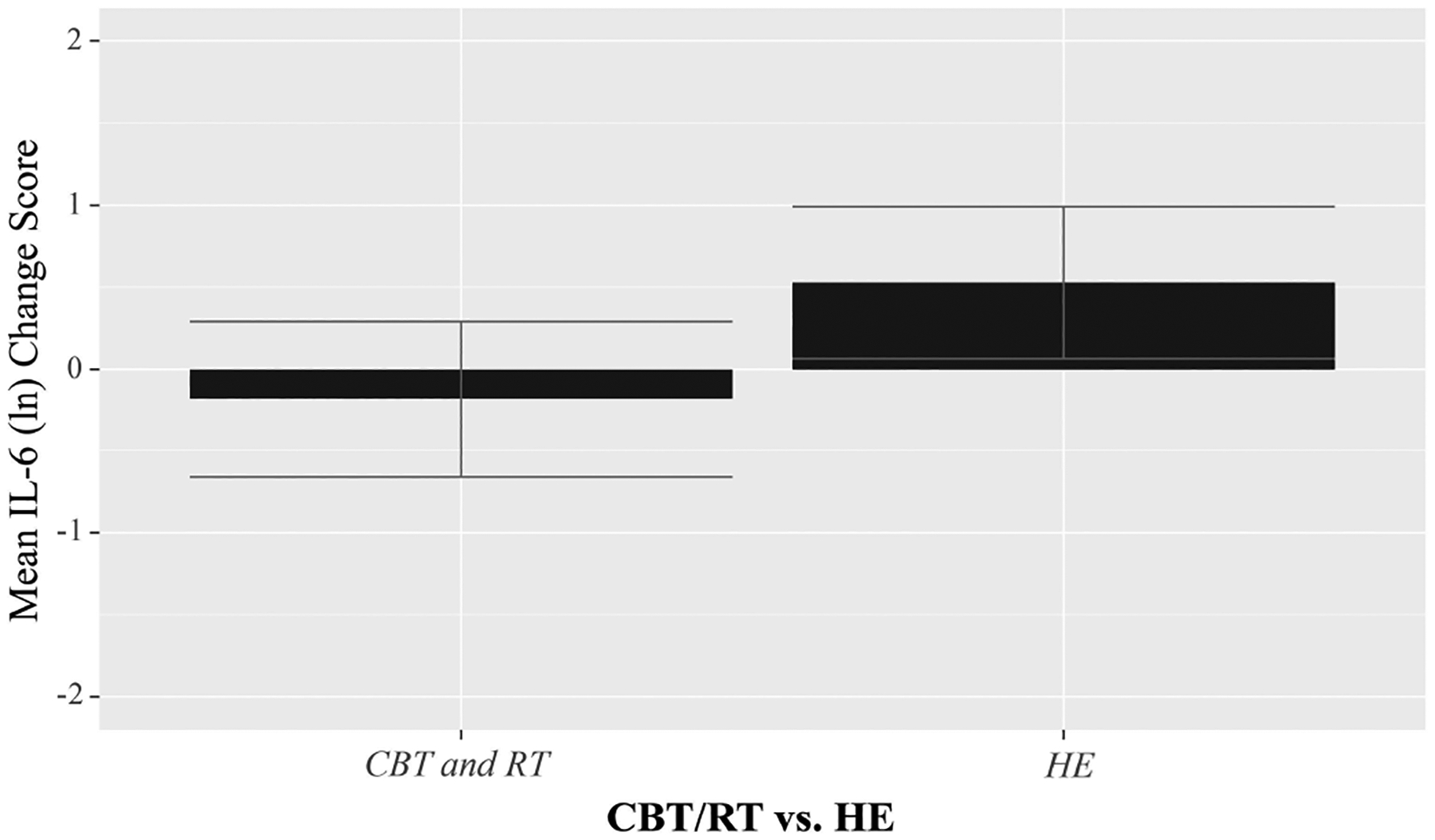
Change in IL-6 pre- to 6-months post-intervention by study condition (CBT/RT vs HE) among overweight and obese women with breast cancer combined. Cytokine levels are log-transformed to achieve normality. Error bars represent 95% confidence intervals. RMANOVA indicated that overweight/obese women receiving CBT/RT had significantly decreased IL-6 levels over time vs those who received HE (F(1,45) = 4.24, *p* = 0.045).

A two condition (CBT/RT vs HE) by two group (OW/OB vs NW) by two timepoint (T1, T3) RMANOVA predicting IL-6 was run. There was no main effect of time in predicting IL-6 (F(1,86) = 0.44, *p* = 0.410), but there was a main effect of BMI (F(1,86) = 9.66, *p* = 0.003), with OW/OB women having greater IL-6 levels across timepoints and conditions. There was a significant three-way time by BMI by condition interaction (F(1,86) = 5.00, *p* = 0.028), indicating moderation. Given the significant three-way interaction, we conducted subgroup analyses by BMI. There was no significant time by condition interaction among NW women, F(1,37) = 0.19, *p* = 0.664), but there was a time by condition interaction among OW/OB women (F(1,45) = 4.24, *p* = 0.045), such that only OW/OB women experienced decreased IL-6 over time when receiving CBT/RT vs HE, as shown by the the mean change score in IL-6 by study condition (CBT/RT vs HE) among OW/OB women (Fig. 3). Supplementary Fig. 1 shows IL-6 change scores by intervention in OW and OB women separately for descriptive purposes. In each case assignment to CBT/RT was associated with a decrease or no increase in IL-6 vs a rise in IL-6 in HE.

An additional two condition (CBT/RT vs HE) by two group (OW/OB vs NW) by two timepoint (T1, T3) RMANOVA predicting IL-1*β* was run. There was no main effect of time (F(1,89) = 0.04, *p* = 0.845) or of BMI (F(1,89) = 2.24, *p* = 0.138) on IL-1*β* when controlling for all other variables in the model. There was no significant three-way time by BMI by condition interaction (F(1,89) = 0.35, *p* = 0.555).

Finally, a two condition (CBT/RT vs HE) by two group (OW/OB vs NW) by two timepoint (T1, T3) RMANOVA predicting TNF-*α* was run. There was no main effect of time (F(1,89) = 0.23, *p* = 0.631) or of BMI (F(1,89) = 0.19, *p* = 0.663) on TNF-*α* when controlling for all other variables in the model. There was no significant three-way time by BMI by condition interaction (F(1,89) = 0.22, *p* = 0.638). See Supplementary Fig. 2 for mean change scores of IL-1*β* and TNF-*α* by condition among OW/OB women.

### 3.4 Examining the Role of Physical Activity Change

There were no significant baseline differences between BMI categories on the self-reported level of MVPA (*t*(132) = 1.75, *p* = 0.082), with OW/OB women having a mean of 0.75 hours of MVPA while NW women had a mean of 1.30 hours in the past week. At follow-up, there was also no difference between groups (*t*(103) = 0.63, *p* = 0.533), with OW/OB women having a mean of 0.57 hours of MVPA while NW women had a mean of 0.67 hours in the past week (Table 1).

In a two condition (CBT/RT vs HE) by two group (OW/OB vs NW) by two timepoint (T1, T3) RMANOVA predicting MVPA controlling for previously stated covariates, there was no main effect of time (F(1,83) = 0.39, *p* = 0.533) or of BMI (F(1,83) = 1.90, *p* = 0.172). There was no significant three-way time by BMI by condition interaction (F(1,83) = 0.01, *p* = 0.920) Finally, we reran the analyses of intervention effects on IL-6 and found that the significant three-way interaction predicting IL-6 change was retained when we controlled for contemporaneous changes in MVPA (F(1,70) = 4.05, *p* = 0.048). Together, this evidence suggests that changes in MVPA did not explain the effects of treatment arm on contemporaneous changes in IL-6 over time.

## 4. Discussion

The present analyses found that both OW and OB women with newly diagnosed BC had significantly greater levels of circulating inflammatory cytokines IL-6 and IL-1*β* than NW patients in the 2–10-week post-surgical period. Importantly, elevated levels of these cytokines are related to poorer health outcomes among women with BC [61]. In addition, we found that BMI category moderated the effect of brief stress management interventions on IL-6 changes over time, such that 5 weeks of stress management intervention of CBT or RT vs a time-matched HE control condition significantly decreased IL-6 in OW/OB but not NW patients. This finding is especially notable given prior literature demonstrating that IL-6 levels negatively predict survival among women with BC [62–64].

We did not find any evidence of a relationship between 5-week stress management, BMI, and the change in inflammatory IL-1*β* or TNF-*α* over the study period. This contrasts with previous findings that a 10-week cognitive behavioral stress management group intervention was associated with reductions in leukocyte *TNFA* gene expression [40]. It is possible that there is a dose-dependent response of stress management, such that a briefer intervention, while potentially more feasible during primary care, is less potent.

A manipulation check demonstrated that women who received the active stress management conditions reported significantly increased perceived stress management skills vs those in the HE control condition, which verified that our active conditions were effectively training participants. Notably, this effect did not differ by BMI. Thus, although both OW/OB and NW women receiving stress management interventions reported significantly greater confidence in their ability to manage stress, unique processes occurred within OW/OB women resulting in decreased IL-6 levels over the study period. It is likely that OW/OB women, who presented with significantly higher IL-6 at baseline, were more able to benefit physiologically from a stress management intervention due to the co-occurring, and potentially interacting, effects of acute stress [38], chronic stress [39], and adiposity [24–29] on neuroendocrine regulation and inflammation. Providing coping and relaxation techniques may have been more impactful in this particularly vulnerable population.

Our analyses were unable to identify the mechanism by which these interventions affected biomarkers within OW/OB women. Given that physical activity is related to inflammatory markers [65] and BMI (i.e., increases in physical activity may reduce BMI), and prior evidence that stress management interventions in other trials may increase engagement in physical activity during BC treatment [66], we tested whether there was an increase in MVPA among OW/OB women participating in stress management groups. However, there was no significant intervention condition by BMI by time interaction effect on MVPA over the study period. Overall, MVPA decreased over the study period across all conditions and BMI categories, likely due to physical limitations associated with adjuvant treatment, and was not a likely explanation for the effects of stress management intervention on cytokine changes. Since there were no differences in adjuvant treatments or other medications received among conditions, we can rule out these as contributing to our results.

Future research should continue to investigate potential mechanisms of change that may explain the relationship between BMI, stress management, and inflammation in BC patients. For example, it is possible stress management may decrease negative self-view and internalized weight stigma among OW/OB women, thereby decreasing stress and inflammation. There is literature suggesting that OW/OB individuals experience significant weight stigma and discrimination in the United States [67–69]. Some OW/OB individuals internalize this weight stigma, thus devaluing themselves based on their weight [70]. Stress management interventions may influence internalized weight stigma by targeting and reappraising cognitions and can provide adaptive coping mechanisms for the related distress. These skills may also allow them to better cope with external weight stigma they face, which have been shown occur frequently in healthcare settings [69], as these women navigate frequent medical appointments for their BC treatment.

In addition, it is possible women who received stress management replaced maladaptive coping techniques with adaptive coping skills. For example, women in stress management conditions may engage in cognitive restructuring (in CBT condition) or mindfulness meditation (in RT condition) when distressed as opposed to emotional eating, a common stress response that may lead to further distress [71] and which may contribute to the development and maintenance of obesity [72]. There is evidence that both behavioral [73] and mindfulness-based psychological interventions [74] decrease emotional eating behaviors. Therefore, it is plausible that in our sample, OW/OB women in the stress management conditions decreased maladaptive emotional eating and increased adaptive coping responses, thereby increasing psychological well-being, and decreasing circulating inflammatory cytokines. Future research should investigate the role of emotional eating in the relationship between BMI, stress management, and inflammation, as well as the role of other eating styles (i.e., mindless vs mindful eating [75], intuitive eating [76], restrictive eating [77]), to add further nuance to these findings.

There are several limitations of the current study to note. First, BMI was calculated through self-report by participants, which may be subject to error. However, there is data demonstrating that self-report and objective weights are generally well-correlated [78,79]. In addition, 30 women who participated in the original trial (16.3%) declined to provide height and weight data and were thus excluded from the current analysis, which may add selection bias to our findings. Further, BMI as a measure of weight status has limitations and may lack specificity in estimating body fat. Due to the nature of the post-hoc analyses, we did not have a longitudinal measure of BMI or weight at follow-up, and therefore cannot know if the change in inflammatory cytokines in the OW/OB women receiving stress management is related to a decrease in weight over the study period. In addition, we did not have a standardized measure of diet or eating styles pre- to post-intervention, which would have added greater nuance to our findings. However, intervention effects on IL-6 held when controlling for contemporaneous changes in physical activity, providing some evidence that our results were not due to changes in energy balance over the study period.

The current analysis is also limited in that adipokines were not collected in addition to cytokines. The relationship between obesity and BC is thought to be likely related to both inflammation, measured here by inflammatory cytokines, as well as deregulation of adipokine secretion [80]. Adiponectin is the most numerous adipokine, has anti-inflammatory properties, and may decrease tumor proliferation, but is significantly decreased in OW/OB women [80–83]. In addition, we did not collect data on the expression of genes that are associated with inflammation and health outcomes in BC, including ErbB2 [84]. Future research should determine whether stress management impacts adiponectin in OW/OB women, and whether specific gene expression may play a role in the relationship between stress and inflammation in OW/OB women with BC.

Despite these limitations, the current study has several strengths. We analyzed immunological, psychological, and behavioral data from a diverse cohort of women (42% Hispanic) with newly diagnosed BC who had participated in a prior RCT. This study adds to the current literature by examining how brief stress management interventions comprising CBSM (CBT- and RT-based) may impact inflammatory biomarkers among BC patients with different BMI levels. This work has important implications for further understanding the biobehavioral mechanisms by which OW/OB women with BC experience worse health outcomes [85,86], and to work towards developing targeted interventions to improve outcomes among this high-risk population.

## 5. Conclusions

OW and OB women with newly diagnosed BC had significantly greater levels of IL-6 and IL-1*β*, which are known to promote poorer health outcomes, as compared to NW women in the post-surgical period. This identifies OW/OB BC patients as a vulnerable group deserving more attention in future research. BMI category also influenced the effect of brief stress management interventions on changes in levels of IL-6, such that stress management (vs a health education control) significantly decreased IL-6 in OW/OB but not NW individuals. Future work should conduct large-scale trials of brief accessible stress management interventions such as these in OW/OB women with BC early in primary treatment and explore the mechanisms by which stress management reduces inflammation and improves future health outcomes in this understudied population.

## Supplementary Material

Supplemental Figure 2

Supplemental Figure 1

Intervention Descriptions

## Figures and Tables

**Table 1.** Demographic and study variables by BMI category.

Normal weight vs Overweight/obese				
Baseline (T1)	NW	OW/OB	Statistic	Diff
	(N = 66)	(N = 83)		
IL-6 (ln)	0.33 (1.34)	1.24 (1.40)	*t*(146) = −4.03	***p* < 0.001** ***
IL-6 (ng/mL)	3.79 (8.73)	15.29 (69.55)	-	-
IL-1*β* (ln)	−0.60 (1.90)	0.26 (1.90)	*t*(147) = −2.76	***p* = 0.007** **
IL-1*β* (ng/mL)	3.58 (10.68)	7.90 (22.77)	-	-
TNF-*α* (ln)	0.41 (0.10)	0.43 (1.12)	*t*(147) = −0.03	*p* = 0.893
TNF-*α* (ng/mL)	2.34 (2.33)	3.84 (13.05)	-	-
Anti-dep.	5 (7.3%)	11 (13.6%)	*χ*^2^(1) = 3.14	*p* = 0.221
Anxiolytic	10 (14.7%)	14 (17.3%)	*χ*^2^(1) = 0.16	*p* = 0.694
Pain med	12 (17.6%)	14 (17.3%)	*χ*^2^(1) = 0.01	*p* = 0 .954
Sleep med	12 (17.6%)	12 (14.6%)	*χ*^2^(1) = 0.25	*p* = 0.616
MVPA (hours)	1.30 (1.94)	0.76 (1.64)	*t*(132) = 1.75	***p* = 0.082** ^t^
Age	53.85 (10.6)	54.71 (10.3)	*t*(108) = −0.42	*p* = 0.676
Income	110.5 (69.7)	118.65 (79.2)	*t*(108) = −0.56	*p* = 0.573
Stage			*χ*^2^(3) = 4.85	*p* = 0.183
Stage 0	13 (19.1%)	13 (15.3%)		
Stage 1	41 (60.3%)	44 (51.8%)		
Stage 2	10 (14.7%)	25 (29.4%)		
Stage 3	4 (5.9%)	3 (3.5%)		
ER+ Status	55 (84.6%)	71 (87.7%)	*χ*^2^(1) = 0.28	*p* = 0.596
PR+ Status	48 (73.8%)	62 (79.5%)	*χ*^2^(1) = 0.64	*p* = 0.425
Mastectomy	34 (50.0%)	43 (50.6%)	*χ*^2^(1) = 0.01	*p* = 0.942
Time f/ surg.	37.3 (25.2)	35.7 (20.2)	*t*(151) = 0.44	*p* = 0.661
Ethnicity			*χ*^2^(2) = 8.91	***p* = 0.021** *
NHW	37 (60.7%)	28 (35.4%)		
Hispanic	21 (34.4%)	43 (54.4%)		
Other	3 (4.9%)	8 (10.1%)		
Follow-up (T3)	NW	OW/OB	Statistic	Diff
	(N = 49)	(N = 54)		
IL-6 (ln)	0.75 (1.44)	1.35 (1.25)	*t*(99) = −2.25	***p* = 0.027** *
IL-6 (ng/mL)	7.49 (19.48)	10.27 (21.51)	-	-
IL-1*β* (ln)	0.04 (2.14)	0.43 (2.16)	*t*(101) = −0.92	*p* = 0.360
IL-1*β* (ng/mL)	7.55 (19.12)	11.45 (36.36)	-	-
TNF-*α* (ln)	0.38 (1.62)	0.43 (1.50)	*t*(101) = −0.14	*p* = 0.893
TNF-*α* (ng/mL)	3.57 (4.03)	3.45 (4.10)	-	-
Anti-dep.	5 (8.9%)	4 (6.7%)	*χ*^2^(1) = 0.21	*p* = 0.649
Anxiolytic	12 (21.4%)	6 (9.8%)	*χ*^2^(1) = 3.01	***p* = 0.083** ^t^
Pain med	10 (17.9%)	3 (5.0%)	*χ*^2^(1) = 4.81	***p* = 0.028** *
Sleep med	11 (20.0%)	9 (15.0%)	*χ*^2^(1) = 0.50	*p* = 0.480
Radiation	9 (16.1%)	11 (18.3%)	*χ*^2^ (1) = 0.11	*p* = 0.746
Chemo	5 (8.9%)	4 (6.7%)	*χ*^2^(1) = 0.21	*p* = 0.651
Reconstr.	14 (24.6%)	22 (36.1%)	*χ*^2^(1) = 1.84	*p* = 0.175
MVPA (hours)	0.67 (0.73)	0.57 (0.73)	*t*(103) = 0.63	*p* = 0.533
Attendance	4.29 (1.17)	3.86 (1.49)	*t*(120) = 1.74	***p* = 0.085** ^t^

Sample size based on participants with cytokine data. CBT, Cognitive Behavioral Therapy; RT, Relaxation Training; HE, Health Education; Anti-dep., antidepressant; MVPA, weekly moderate/vigorous physical activity; med, medicine; NHW, non-Hispanic White; chemo, chemotherapy; time f/ surg., days from surgery until T1; reconst, reconstructive surgery. Income measured in thousands.

t *p* < 0.10,

* *p* < 0.05,

** *p* < 0.01,

*** *p* < 0.001.

**Table 2.** Demographic and study variables by study condition within BMI category.

	Normal weight (CBT/RT vs HE) (N = 66)				Overweight/obese (CBT/RT Vvs HE) (N = 83)			
Baseline (T1)	CBT/RT	HE	Statistic	Diff.	CBT/RT	HE	Statistic	Diff.
	(N = 42)	(N = 24)			(N = 56)	(N = 27)		
IL-6 (ln)	0.29 (1.25)	0.39 (1.50)	*t*(64) = −0.26	*p* = 0.792	1.34 (1.36)	0.97 (1.47)	*t*(80) = 1.20	*p* = 0.250
IL-6 (ng/mL)	2.75 (4.12)	5.62 (13.40)	-	-	18.32 (83.0)	8.77 (21.65)	-	-
IL-1*β* (ln)	−0.33 (1.78)	1.08 (2.04)	*t*(64) = 1.56	*p* = 0.124	0.51 (1.78)	−0.25 (2.07)	*t*(81) = 1.73	*p* = 0.108
IL-1*β* (ng/mL)	3.00 (5.59)	4.58 (16.27)	-	-	8.53 (25.00)	6.60 (17.71)	-	-
TNF-*α* (ln)	0.57 (1.04)	0.12 (0.86)	*t*(64) = 1.79	***p* = 0.078** ^t^	0.33 (1.10)	0.64 (1.14)	*t*(81) = −1.21	*p* = 0.231
TNF-*α* (ng/mL)	2.75 (2.59)	1.63 (1.59)	-	-	4.00 (15.68)	3.51 (4.08)	-	-
Anti-dep.	5 (11.6%)	0 (0%)	*χ*^2^(1) = 3.14	*p* = 0.150	9 (16.3%)	2 (7.7%)	*χ*^2^(1) = 1.13	*p* = 0.489
Anxiolytic	6 (14.0%)	4 (16.0%)	*χ*^2^(1) = 0.05	*p* = 0.818	11 (20%)	3 (11.1%)	*χ*^2^(1) = 1.01	*p* = 0.369
Pain med	9 (20.9%)	3 (12.0%)	*χ*^2^(1) = 0.87	*p* = 0.352	9 (16.3%)	5 (12.0%)	*χ*^2^(1) = 0.10	*p* = 0.750
Sleep med	7 (16.3%)	5 (20%)	*χ*^2^(1) = 0.15	*p* = 0.700	9 (16.3%)	3 (11.1%)	*χ*^2^(1) = 0.40	*p* = 0.527
MVPA (hours)	1.40 (1.82)	1.13 (2.14)	*t*(58) = 0.53	*p* = 0.597	0.73 (1.63)	0.81 (1.69)	*t*(72) = −0.21	*p* = 0.831
Age	52.88 (10.6)	55.52 (10.5)	*t*(66) = −0.99	*p* = 0.326	56.66 (10.0)	54.74 (8.3)	*t*(83) = 0.87	*p* = 0.390
Income	94.84 (50.8)	137.43 (88.7)	*t*(66) = −2.53	***p* = 0.014** *	99.22 (69.9)	110.34 (74.4)	*t*(83) = −0.67	*p* = 0.505
Stage			*χ*^2^(3) = 3.29	*p* = 0.349			*χ*^2^(3) = 1.66	*p* = 0.647
Stage 0	11 (25.6%)	2 (8.0%)			7 (12.1%)	6 (22.2%)		
Stage 1	24 (55.8%)	17 (68.0%)			32 (55.2%)	12 (44.4%)		
Stage 2	6 (14.0%)	4 (16.0%)			17 (29.3%)	8 (29.6%)		
Stage 3	2 (4.7%)	2 (8.0%)			2 (3.4%)	1 (3.7%)		
ER+ Status	35 (85.0%)	20 (80.0%)	*χ*^2^(1) = 0.67	*p* = 0.415	51 (91.1%)	20 (80%)	*χ*^2^(1) = 1.96	*p* = 0.271
PR+ Status	30 (75.0%)	18 (71.0%)	*χ*^2^(1) = 0.07	*p* = 0.749	43 (78.2%)	19 (82.6%)	*χ*^2^(1) = 0.20	*p* = 0.659
Mastectomy	22 (51.2%)	12 (48.0%)	*χ*^2^(1) = 0.06	*p* = 0.801	30 (51.7%)	13 (48.1%)	*χ*^2^(1) = 0.09	*p* = 0.759
Time f/ surg.	39.2 (29.6)	34.0 (15.0)	*t*(66) = 0.81	*p* = 0.419	36.0 (21.5)	35.1 (17.5)	*t*(83) = 0.18	*p* = 0.857
Ethnicity			*χ*^2^(2) = 2.20	*p* = 0.334			*χ*^2^(2) = 0.09	*p* = 0.958
NHW	21 (56.8%)	16 (66.7%)			19 (35.8%)	9 (34.6%)		
Hispanic	15 (40.5%)	6 (25.5%)			29 (54.7%)	14 (53.8%)		
Other	1 (2.7%)	2 (8.3%)			5 (9.4%)	3 (11.5%)		
Follow-up (T3)	CBT/RT	HE	Statistic	Diff.	CBT/RT	HE	Statistic	Diff.
	(N = 31)	(N = 18)				(N = 21)		
IL-6 (ln)	0.92 (1.57)	0.45 (1.18)	*t*(46) = 1.09	*p* = 0.280	1.47 (1.15)	1.14 (1.39)	*t*(51) = 0.94	*p* = 0.353
IL-6 (ng/mL)	10.32 (24.2)	2.77 (2.91)	-	-	10.65 (21.5)	9.64 (22.11)	-	-
IL-1*β* (ln)	0.19 (2.28)	−0.21 (1.93)	*t*(47) = 0.62	*p* = 0.555	0.60 (1.93)	0.15 (2.50)	*t*(52) = 0.75	*p* = 0.459
IL-1*β* (ng/mL)	10.11 (23.5)	3.15 (4.82)	-	-	6.46 (9.70)	19.28 (56.98)	-	-
TNF-*α* (ln)	0.40 (1.74)	0.36 (1.44)	*t*(47) = 0.08	*p* = 0.935	0.34 (1.34)	0.56 (1.75)	*t*(52) = −0.51	*p* = 0.611
TNF-*α* (ng/mL)	3.88 (4.37)	3.05 (3.42)	-	-	2.71 (2.72)	4.62 (5.52)	-	-
Anti-dep.	4 (11.1%)	1 (4.8%)	*χ*^2^(1) = 0.72	*p* = 0.397	2 (5.1%)	2 (9.0%)	*χ*^2^(1) = 0.33	*p* = 0.567
Anxiolytic	7 (19.4%)	5 (23.8%)	*χ*^2^(1) = 0.11	*p* = 0.737	5 (12.8%)	1 (4.5%)	*χ*^2^(1) = 1.09	*p* = 0.297
Pain med	4 (11.1%)	6 (28.6%)	*χ*^2^(1) = 2.63	*p* = 0.105	2 (5.1%)	1 (4.5%)	*χ*^2^(1) = 0.02	*p* = 0.902
Sleep med	8 (22.2%)	3 (14.3%)	*χ*^2^(1) = 0.69	*p* = 0.405	5 (12.8%)	4 (18.2%)	*χ*^2^(1) = 0.28	*p* = 0.599
Radiation	6 (16.7%)	3 (14.3%)	*χ*^2^(1) = 0.06	*p* = 0.812	8 (20.5%)	3 (13.6%)	*χ*^2^(1) = 0.45	*p* = 0.502
Chemo	5 (13.9%)	0 (0%)	*χ*^2^(1) = 3.20	***p* = 0.074** ^t^	3 (7.7%)	1 (4.5%)	*χ*^2^(1) = 0.23	*p* = 0.634
Reconstr.	10 (27.8%)	4 (19.0%)	*χ*^2^(1) = 0.55	*p* = 0.460	15 (38.5%)	7 (31.8%)	*χ*^2^(1) = 0.27	*p* = 0.604
MVPA (hours)	0.60 (0.73)	0.76 (0.74)	*t*(49) = −0.78	*p* = 0.442	0.54 (0.77)	0.65 (0.68)	*t*(52) = −0.56	*p* = 0.578
Attendance	4.22 (1.27)	4.47 (0.915)	*t*(49) = −0.68	*p* = 0.503	3.72 (1.57)	4.19 (1.25)	*t*(69) = −1.22	*p* = 0.226

Sample size based on participants with cytokine data. CBT, Cognitive Behavioral Therapy; RT, Relaxation Training; HE, Health Education; Anti-dep., antidepressant; MVPA, weekly hours of moderate/vigorous physical activity; med, medicine; NHW, non-Hispanic White; chemo, chemotherapy; time f/ surg., days from surgery until T1; reconst, reconstructive surgery. Income measured in thousands.

t *p* < 0.10,

* *p* < 0.05,

** *p* < 0.01,

*** *p* < 0.001.

## Abbreviations

ACS: American Cancer Society
ANOVA: Analysis of variance
BC: Breast cancer
BMI: Body mass index
CBSM: Cognitive-Behavioral Stress Management
CBT: Cognitive behavioral therapy
CDC: Center for Disease Control and Prevention
DFS: Disease-free survival
ELISA: Enzyme-linked immunosorbent assay
ER/PR: Estrogen receptor/Progesterone receptor
HE: Health education
IL-1*β*: Interleukin-1 beta
IL-6: Interleukin-6
LSD: Least Squared Differences
MVPA: Moderate and vigorous physical activity
NCI: National Cancer Institute
NHW: Non-Hispanic White
NIH: National Institutes of Health
NW: Normal weight
OW/OB: Overweight and obese
PSMS: Perceived stress management skills
RMANOVA: Repeated Measures Analysis of variance
RT: Relaxation training
SD: Standard deviation
SPSS: Statistical Package for the Social Sciences
T1: Timepoint 1: Baseline timepoint
T2: Timepoint 2: ~6-weeks post baseline
T3: Timepoint 3: Six months after randomization
TNF-*α*: Tumor necrosis factor alpha
WHO: World Health Organization
