# Supplementary figures and images for: Brief Post-Surgical Stress Management Reduces Pro-Inflammatory Cytokines in Overweight and Obese Breast Cancer Patients Undergoing Primary Treatment
Source: Front Biosci (Landmark Ed). Author manuscript; Available in PMC 2022 Jul 1. (PMC9248770; doi:10.31083/j.fbl2705148)

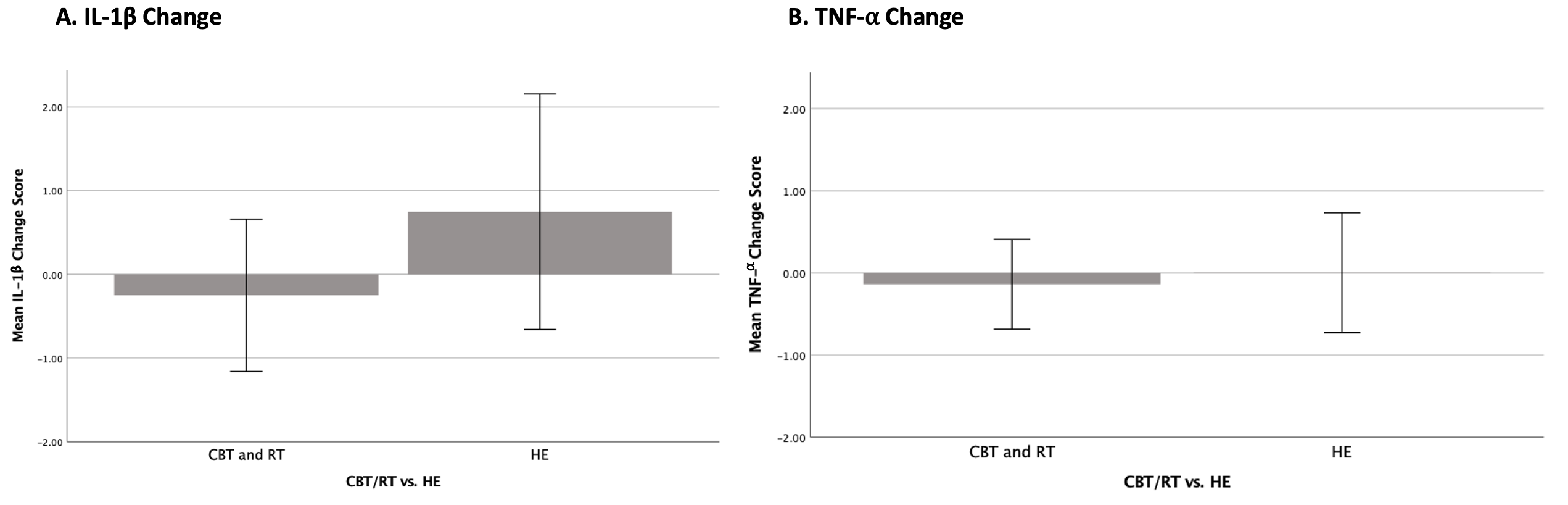

Supplement: Supplemental Figure 2 [file NIHMS1818401-supplement-Supplemental_Figure_2.png]

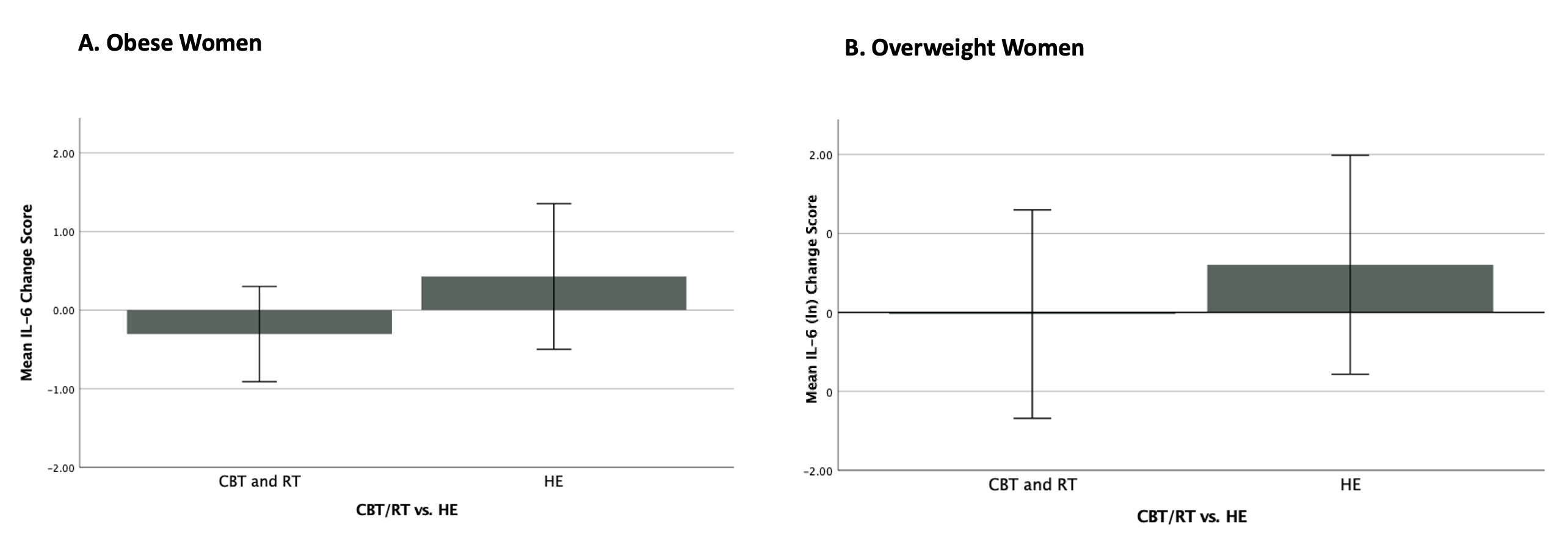

Supplement: Supplemental Figure 1 [file NIHMS1818401-supplement-Supplemental_Figure_1.png]
