# Supplementary material for: Brief Post-Surgical Stress Management Reduces Pro-Inflammatory Cytokines in Overweight and Obese Breast Cancer Patients Undergoing Primary Treatment
Source: Front Biosci (Landmark Ed). Author manuscript; Available in PMC 2022 Jul 1. (PMC9248770; doi:10.31083/j.fbl2705148)
Supplement: Intervention Descriptions [file NIHMS1818401-supplement-Intervention_Descriptions.docx]

**Weekly Outline of Interventions**

1. Cognitive Behavioral Therapy

Session 1: Stress symptoms and appraisals

Session 2: Cognitive distortions and reappraisals

Session 3: Coping; acceptance and softening

Session 4: Social support and anger management

Session 5: Assertiveness and review

*Women were assigned weekly out-of-session homework practices (e.g., thought monitoring worksheets) to practice skills learned in session

1. Relaxation Training

Session 1: Rationale for relaxation; progressive muscle relaxation

Session 2: Progressive muscle relaxation; mental imagery

Session 3: Passive progressive muscle relaxation; mental imagery

Session 4: Light imagery

Session 5: Mindfulness meditation

*Women were provided audio recordings of each relaxation practice and instructed to practice daily between sessions

1. Health Education Control

Session 1: General overview of breast cancer and available resources

Session 2: Treatment options for breast cancer

Session 3: Side effect management

Session 4: Healthy lifestyle: physical activity and nutrition

Session 5: Quality of life and planning for the future
